# FOXG1 Contributes Adult Hippocampal Neurogenesis in Mice

**DOI:** 10.3390/ijms232314979

**Published:** 2022-11-29

**Authors:** Jia Wang, Hong-Ru Zhai, Si-Fei Ma, Hou-Zhen Shi, Wei-Jun Zhang, Qi Yun, Wen-Jun Liu, Zi-Zhong Liu, Wei-Ning Zhang

**Affiliations:** 1 Department of Neurology, The Fourth Affiliated Hospital of Jiangsu University, Zhenjiang 212001, China; 2 Department of Laboratory Medicine, School of Medicine, Jiangsu University, Zhenjiang 212013, China

**Keywords:** Alzheimer’s disease, FOXG1, cell cycle, neural stem cell, hippocampus

## Abstract

Strategies to enhance hippocampal precursor cells efficiently differentiate into neurons could be crucial for structural repair after neurodegenerative damage. FOXG1 has been shown to play an important role in pattern formation, cell proliferation, and cell specification during embryonic and early postnatal neurogenesis. Thus far, the role of FOXG1 in adult hippocampal neurogenesis is largely unknown. Utilizing CAG-loxp-stop-loxp-Foxg1-IRES-EGFP (*Foxg1*^fl/fl^), a specific mouse line combined with CreAAV infusion, we successfully forced FOXG1 overexpressed in the hippocampal dentate gyrus (DG) of the genotype mice. Thereafter, we explored the function of FOXG1 on neuronal lineage progression and hippocampal neurogenesis in adult mice. By inhibiting p21^cip1^ expression, FOXG1-regulated activities enable the expansion of the precursor cell population. Besides, FOXG1 induced quiescent radial-glia like type I neural progenitor, giving rise to intermediate progenitor cells, neuroblasts in the hippocampal DG. Through increasing the length of G_1_ phase, FOXG1 promoted lineage-committed cells to exit the cell cycle and differentiate into mature neurons. The present results suggest that FOXG1 likely promotes neuronal lineage progression and thereby contributes to adult hippocampal neurogenesis. Elevating FOXG1 levels either pharmacologically or through other means could present a therapeutic strategy for disease related with neuronal loss.

## 1. Introduction

The presence of adult neuronal stem cells (aNSCs) capable of generating new neurons throughout life provides adult mammals with an exceptional level of brain plasticity [1]. Accumulated behavioral evidence suggests that the hippocampus plays a critical role in learning and memory [2], which depend on functional and structural changes, such as synaptic remodeling occurring in the hippocampus [3]. The discovery of a de novo production of neurons in the adult dentate gyrus (DG) has increased the possibility of a new form of plasticity that could sustain memory processes [4]. Hence, the development of new therapeutic strategies, which could induce neurogenesis and generate postmitotic neurons to promote the regenerative and recovery process of neurodegenerative damage, has caused extensive concern. 

Forkhead-box gene 1 (*Foxg1*), is a member of the forkhead-box family of transcription factors that is strongly expressed in the cerebral cortex, telencephalon, olfactory epithelial cells, and other neural and sensory tissues [5]. The purported roles of *Foxg1* include but are not limited to (1) promoting the overgrowth of neural progenitor cells (NPCs) [6], (2) inducing differentiation of normal NSCs in the cerebral cortex during development, (3) and regulating early cortical cell fate [7]. In light of that, most efforts have been devoted to explain the cellular basis of FOXG1 in pattern formation, cell proliferation, and cell specification during embryonic and early postnatal neurogenesis [8]. Thus far, the role of *Foxg1* in adult hippocampal neurogenesis is largely unknown. Based on the reports that FOXG1 increases the population of NPCs likely through inhibiting the expression of p21WAF1/cyclin-dependent kinase interacting protein 1 protein during embryonic development [5], together with the finding that FOXG1 protein is mainly expressed in a subpopulation of adult SGZ cells of the hippocampal DG [9], we are tempted to speculate that FOXG1 possibly contributes to neuronal precursors, with the possibility of proliferation, redifferentiation, and generation of new neurons, providing adult mammals with an exceptional level of brain plasticity. Thus, the overarching goal of the research is to elucidate the functions of FOXG1 on neuronal lineage progression with a particular focus on five developmental stages during adult hippocampal neurogenesis: (1) activation of quiescent NSCs (qNSCs) in the SGZ; (2) enlargement of precursor cell pool; (3) generation of newborn neuroblasts; (4) promotion of lineage-committed cells exiting the cell cycle; (5) maturation of adult-born neurons. 

## 2. Materials and Methods

### 2.1. Animals

All of the genotype mice were housed with 12 h light: dark cycle, free access to water and food, at 22 ± 2 °C. They were maintained in the animal center of Jiangsu University (Zhenjiang, China) in compliance with the Guide for the Care and Use of Laboratory Animals (NIH Publication No. 8023, revised 1978). Experiments protocols were approved by the Committee on Humane Use of Animals at Jiangsu University (SYXK2018-0053, permited: 2018). All experiments were conducted in 4-month-old male mice. 

#### 2.1.1. *Foxg1* Transgenic Mice

CAG-loxp-stop-loxp-Foxg1-IRES-EGFP (*Foxg1*^fl/fl^) mice were kindly provided by Dr. Chunjie Zhao (Medical School of Southeast University, China). Genotypes of the offspring were determined by PCR analysis using primers (5′-AAG GAC GAC GGC AAC TAC AAG-3′, and 5′-GGC GGT CAC GAA CTC CA-3′) to amplify a 378 bp *Foxg1* fragment.

#### 2.1.2. Forced Expression of FOXG1 in Adult Hippocampal DG of the *Foxg1*^fl/fl^ Mice

To force FOXG1 overexpression, the Vgc of rAAV2/9-CMV-Cre-WPRE-pA (CreAAV) was microinjected into hippocampal DG of the *Foxg1* genotype mice. Specifically, *Foxg1*^fl/fl^ mouse was anesthetized with ketamine/xylazine (73/11.6 mg/kg, i.p) and placed in a stereotaxic frame (RWD Life Science, Shenzhen, China) with a mouse adapter. The tip of a pulled glass pipette diameter) was inserted to stereotaxic coordinates AP: −2.06 mm; ML: ±1.70 mm; DV: −2.0 mm, relative to bregma according to the procedures described by our lab [10]. Viral vector of CreAAV suspension in a volume of 0.5 μL was microinjected into each side of the hippocampus of genotype mouse using 2 μL bursts from a Nanoliter 2000 injector (World Precision Instruments, Shanghai, China). This corresponded to 2 × 10^12^ viral genome copies (vgc). 

### 2.2. Histology

Mice were perfused before brains were removed and subsequently embedded in paraffin. Then, the embedded samples were cut into 6 μm coronal sections on a paraffin microtome. The sections were examined to verify and draw the locations of injector tip placement onto plates taken from the atlas of Paxinos and Watson. If the infusion site fells outside targeted area, then its data were excluded from final statistical analyses.

### 2.3. Western Blot Analysis

Mice (*n* = 6 in each group) were decapitated, the hippocampus were immediately dissected, frozen and stored at −80 °C. Tissue samples were lysed and western blots were performed as previously described [11]. Primary antibodies include GFP (1:1000, Beyotime, cat # AG281, Shanghai, China), Glial fibrillary acidic protein (GFAP) (1:1000, Wanleibio, cat # WL0836, Shenyang, China), Flag (1:1000, abm, cat # G188, Zhenjiang, China), Oligo2 (1:1000, Wanleibio cat # WL04942, Shenyang, China), NeuN (1:1000, Wanleibio, cat # WL03099, Shenyang, China), doublecortin (Dcx) (1:1000, Proteintech, cat # 13925-1-AP, Rosemont, USA), GAPDH (1:5000, Boster, cat # BM1623, Wuhan, China) and β-Tubulin (1:5000, Beidibio, Nanjing, China). HRP-conjugated secondary antibodies purchased from Immunoway Biotechnology Company (goat anti mouse cat # RS0001 and goat anti rabbit cat # RS0002, Plano, TX, USA) were used to probe these blots. Protein visualization was carried out using an enhanced chemiluminescen (ECL, Meilunbio, Dalian, China). The signal intensity was obtained by densitometric scanning.

### 2.4. Immunohistochemistry 

After anesthetization, perfusion, and fixation, the brains of the mice (*n =* 6 in each group) were embedded in paraffin. Coronal paraffin sections (6 μm) from the hippocampus of the mouse brain were used to investigate. Brain sections were immunolabeled with antibodies against GFAP (1:200, Wanleibio, cat # WL0836, ShenYang, China), Tbr2 (1:200, Bioss, cat # bs-11331R, Beijing, China), NeuN (1:200, Bioss, cat # bs-10394R, Beijing, China) or Oligo2 (1:200, Bioss, cat # bs-11194R, Beijing, China). Immunohistochemistry secondary antibodies were purchased from Boster Biological Technology Company (HRP Vision IgG antibody cat # SV0001 and cat # SV0002, Wuhan, China). After washing, DAB (Boster, cat # AR1027, Wuhan, China) was used for dyeing and hematoxylin was used for retaining. The sections were dehydrated in a graded series of ethanol and xylene and mounted using non-aqueous mounting medium. Selected images were captured using a SPOT camera and software system (Diagnostic Instruments, Sterling Heights, MI, USA) attached to a Leica DM5000B microscope (Leica, Bannockburn, IL, USA) with 4 × objectives.

### 2.5. Immunofluorescence

Briefly, 6-μm-thick sections from the hippoampal DG of the *Foxg1* genotype mice (*n =* 6 in each group) were prepared for immunofluorescence analyses as described previously [12,13]. To visualize protein expression, sections were first incubated with primary antibodies, including rabbit anti-GFAP (1:200, Wanleibio, cat # WL0836, Shenyang, China), mouse anti-GFAP (1:200, Proteintech, cat# 60190-1-lg, Rosemont, USA), EGFP (1:200, Beyotime, cat # AG281, Shanghai, China), brain lipid binding protein (BLBP) (1:200, Proteintech, cat # 51010-1-AP, Rosemont, USA), Proliferating Cell Nuclear Antigen (PCNA) (1:250, Proteintech, cat # 10205-2-AP, Rosemont, USA), Tbr2 (1:200, Bioss, cat # bs-11331R, Beijing, China), Oligo2 (1:200, Bioss, cat # bs-11194R, Beijing, China), NeuN (1:1200, Bioss, cat # bs-10394R, Beijing, China) and Dcx (1:200, Proteintech, cat # 13925-1-AP, Rosemont, USA). Immunofluorescence secondary antibodies, Dylight 488 (cat # BA1126) and CY3 (cat # BA1032), were obtained from Boster (Wuhan, China). DAPI (KeyGen Biotech, cat # KGA215-50, Shanghai, China) was used to stain the nuclei. After being severally rinsed in PBT, the sections were mounted using antifade mounting medium (Beyotime, Shanghai, China) and fluorescence was visualized using a fluorescence microscope (BX41, Olympus, Tokyo, Japan). 

### 2.6. Cell Cycle Analysis 

Unless stated otherwise, all culture media were purchased from Invitrogen (Carlsbad, CA, USA) and reagents and chemicals were purchased from Sigma-Aldrich (St. Louis, MO, USA). 

#### 2.6.1. Establishment and Evaluation of FUCCI System

The FUCCI system was used to visualize the cell-cycle phase in individual 293T cells. Plasmids expressing monomeric Kusabira Orange2 (mKO2)-hCdt1 (orange-red fluorescent protein) during G_1_ phase or monomeric Azami Green (mAG)-hGem (green-yellow fluorescent protein) during G_2_/M phase were obtained from the miaolingbio (Wuhan, China). FUCCI plasmids were packaged into AAV vectors (BOB-EF1-FastFUCCI-Puro Lentivirus) and used to infect the 293T cells. FUCCI-AAV infected cells were transfected with control plasmid (pEnCMV, Miaoling Bio, Wuhan, China) or plasmid encoding *Foxg1* (pECMV-Foxg1-m-FLAG, miaolingbio, Wuhan, China) (*n =* 6 in each group) using Lipofectamine 2000 reagent (cat # SCSP-502, Life Technologies, Shanghai, China) for 48 h. Fluorescence was visualized using a FluoView FV1000 confocal laser microscope (BX41, Olympus, Tokyo, Japan) to evaluate the FUCCI traced cell-cycle progression. 

#### 2.6.2. One-Dimensional DNA Measurement of G_1_-Phase Exit 

One-dimensional DNA measurement was performed to analyze cell cycle distribution. Briefly, 750,000 N_2_A cells were seeded into 6-well plates, transfected with *vector* or *Foxg1* plasmids for 48 h (*n =* 6 in each group), the cells were trypsinized, centrifuged at 2000 rpm for 5 min. After washing with PBS and treatment with 70% cold ethanol overnight, the RNase A and propidium iodide mixture were added into the cells for 30 min at room temperature in dark, then 1×10^6^ cells were analyzed with CytExpert (Beckman Coulter, Indianapolis, IN, USA).

#### 2.6.3. Two-Dimensional DNA and RNA Analyses of Cell Cycle Redistribution

Two-dimensional (multi-parameter) DNA and RNA analyses were performed to evaluate cell cycle exit [14]. Cells were transfected with *vector* or *Foxg1* plasmids for 48 h (*n =* 6 in each group). After fixation, cell pellets were resuspended with 400 μL DNA staining solution containing 2 μg/mL Hoechst 33342 (Beyotime Biotechnology, cat # C1002, Shanghai, China), and incubated at room temperature for 30 min. Afterwards, 4 μg/mL Pyronin Y (Sigma, cat # 213519, St. Louis, MO, USA) was added to stain RNA. After washing, nucleic acid contents were analyzed using a CytExpert flow cytometer, and data were evaluated with FlowJo 7.6.5 software (LLC, Ashland, OR, USA). Gates were applied and cell cycle phases were determined according to Darzynkiewicz and Shapiro [14].

#### 2.6.4. Flow Cytometry Detection of p21 Expression 

The effect of p21^cip1^-mediated cell cycle regulation was measured with a cell cycle detection kit (KeyGen Biotech, cat # KGA511, Shanghai, China). Briefly, N_2_A cells were transfected with *vector* or *Foxg1* plasmids for 48 h (*n =* 6 in each group). After incubation with an antibody against p21 (1: 200, Bioss, cat # bs-10129R-FITC, Beijing, China) for 2 h, the cells were resuspended in 0.5 mL buffer containing RNase A (50 μL) and propidium iodide (450 μL) at 37 °C for 30 min in the dark and analyzed by flow cytometry within 1 h. Cell cycle and p21 expression were determined with CytExpert software by Beckman Coulter.

### 2.7. Hippocapal aNSC Cultures

#### 2.7.1. Sample Harvesting

Four-month-old adult (*Foxg1*^fl/fl^ and *Foxg1*^fl/fl^-CreAAV) mice (*n =* 6 in each group) were sacrificed after anesthetization. aNSCs were isolated as described by Hu Y.D [15]. The skulls were dissected, the brain hemisphere was removed and transferred into pre-cooled PBS. Under an anatomic microscope, the hippocampal DG was carefully microdissected while the molecular layer and all ventricular zone cotaminants were removed. 

#### 2.7.2. Preparation of Single Cell Suspension and Cell Inoculation

Hippocampal tissues were sheared into 1 mm^3^ sized tissue blocks. Cell suspension was prepared as the method described by Hu et al. [15]. Cell density was adjusted to 5 × 10^5^/mL. The cells were inoculated onto the culture plates with cuture media including Neurobasal^TM^-A (Gibco, cat # 10888022, Waltham, MA, USA), B-27 Supplement Minus Vitamin A (Gibco, cat # 12587001, Waltham, MA, USA), EGF Protein (R&D Systems, cat # 2028-EG-200, Minneapolis, MN, USA), and FGF Protein (R&D Systems, cat # 3139-FB-025/CF, Minneapolis, MN, USA). At day 5 post-culture, the diameter of neurospheres was commonly~100 μm and subculturing was performed according to the methods described by Hu et al. [15].

#### 2.7.3. Morphological Observation

During the primary and secondary culture of cells derived from the hippocampus of *Foxg1* genotype mice, inverted phase contrast microscopy (Leica Microsystems CMS GmbH, Wetzlar, Germany) was employed to observe and record the morphology and growth of aNSCs. 

### 2.8. Statistical Analyses

For all immunolabeling studies, assessment of immunoreactive levels was performed using the Image-Pro Plus program (Media Cybernetics, Rockville, MD, USA). For each animal, digital images from 6 brain sections were analyzed to estimate the average number of immunolabeled cells per unit area (mm^2^). Unpaired Student’s t-test was used for imaging and immunoblotting data (Graph Pad Prism version 6.0, La Jolla, CA, USA). Differences were considered significant at *p* < 0.05. All values are presented as mean ± standard error of the mean.

## 3. Results

### 3.1. FOXG1 Is Forced Overexpressing in the Hippocampal DG of the Foxg1^fl/fl^ Mouse by Infusing CreAAV

**Figure 1:**
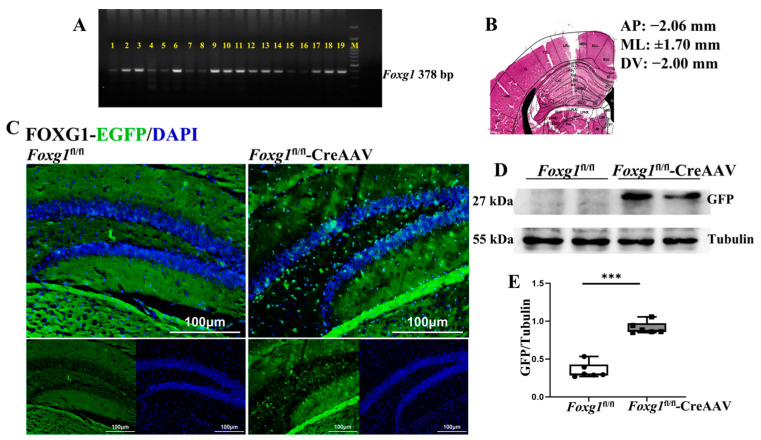
rAAV2/9 forces FOXG1 overexpressed in adult hippocampal DG of the *Foxg1* genotype mice. (**A**) Genotypes of the *Foxg1*^fl/fl^ offspring were determined by PCR analysis using the primers for *Foxg1*. Lanes of 2–3, 6, 9–14, 17–19 represent *Foxg1*^fl/fl^ genotype. The 378-bp band results from amplification of the *Foxg1* allele. (**B**) Location of infusion sites is within the hippocampus area of genotype mice. Photomicrograph represents cresyl violet stained coronal sections from the brain of a mouse with representative placement in the dentate gyrus (DG) region of the hippocampus. (**C**) Expression patterns of FOXG1 were assessed with an antibody against EGFP in the hippocampal DG of mice. Green = EGFP-labeled FOXG1; Blue = DAPI. Scale bars = 100 μm. (**D**) Hippocampal lysates of two genotype mice were immunoblotted using an antibody against GFP. Tubulin was used as loading control. (**E**) Values are expressed as means ± S. E. M. For each group, *n =* 6/group. *** *p* < 0.001 for noted differences between *Foxg1*^fl/fl^ and *Foxg1*^fl/fl^-CreAAV groups.

To address the functions of FOXG1 in adult DG NSCs during hippocampal neurogenesis, we forced FOXG1 overexpression in the hippocampal DG of adult *Foxg1*^fl/fl^ mice by infusing CreAAV (Figure 1A,B), then traced activated FOXG1 with an EGFP tag (Figure 1C). As shown in Figure 1C–E, a strong expression of EGFP-labeled FOXG1 was observed in the hippocampal DG of *Foxg1*^fl/fl^ mouse three weeks after infusion.

### 3.2. FOXG1 Enlarges aNSC Pool and Promotes Their Activation in the Hippocampal DG of Foxg1 Genotype Mice

**Figure 2:**
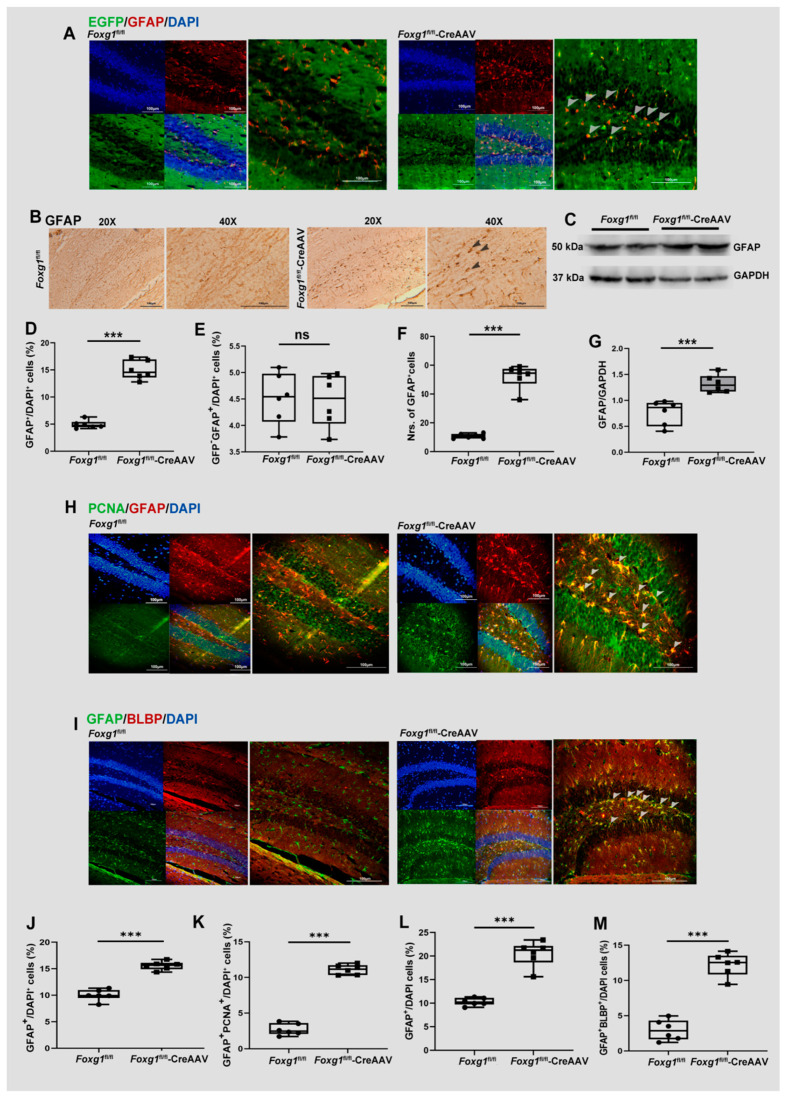
FOXG1 increases the amount of neuronal stem cells (aNSCs) in adult hippocampal DG of the *Foxg1* genotype mice and promotes qNSCs giving rise to activated aNSCs. (**A**) Expression patterns of GFAP-expressing qNSCs in the hippocampal DG of *Foxg1* genotype mice were assessed with immunofluorescence. Red = GFAP; Green = EGFP-labeled FOXG1; Blue = DAPI. Scale bars = 100 μm. Arrow heads: newly generated qNSCs with FOXG1 activation. (**D**) Levels of total qNSCs in adult hippocampus are showed as %GFAP^+^/DAPI^+^ cells. (**E**) Levels of endogenous qNSCs in adult hippocampus are showed as %GFP^−^GFAP^+^/DAPI^+^ cells. (**B**) Expression patterns and (**F**) qualification of total qNSCs in adult hippocampus were studied with immunohischemisitry using an antibody against GFAP. Scale bars = 100 μm. (**C**) Hippocampal lysates of the two genotype mice were immunoblotted using an antibody against GFAP. (**G**) Qualification of GFAP expression is illustrated by bar graph. GAPDH was used as the loading control. (**H**) Expression patterns of proliferated qNSCs in the hippocampal DG of *Foxg1* genotype mice were assessed with immunofluorescence. Red = GFAP; Green = Proliferating Cell Nuclear Antigen (PCNA); Blue = DAPI. Scale bars = 100 μm. Arrow heads: proliferated qNSCs. (**J**) Levels of qNSCs in adult hippocampus are showed as %GFAP^+^/DAPI^+^ cells. (**K**) Levels of proliferated qNSCs in adult hippocampus are showed as %GFAP^+^PCNA^+^/DAPI^+^ cells. (**I**) Expression patterns of activated aNSCs in the hippocampal DG of *Foxg1* genotype mice were assessed with immunofluorescence. Red = brain lipid binding protein (BLBP); Green = GFAP; Blue = DAPI. Scale bars = 100 μm. Arrow heads: activated aNSCs. (**L**) Levels of aNSCs in adult hippocampus are showed as %GFAP^+^/DAPI^+^ cells. (**M**) Levels of activated aNSCs in adult hippocampus are showed as %GFAP^+^BLBP^+^/DAPI^+^ cells. Values are expressed as means ± S. E. M. *n =* 6/group. Significant levels set at *** *p* < 0.001 noted difference between *Foxg1*^fl/fl^ and *Foxg1*^fl/fl^-CreAAV animals. ns—not significant.

Glial fibrillary acidic protein (GFAP)-expressing radial glia-like cells represent qNSCs [16]. Adult hippocampal neurogenesis originates from a population of precursor cells with glial properties [16]. A subset of these shows morphological and antigenic characteristics of radial glia [16]. Their cell body is found in the SGZ and the process extends into the molecular layer [16]. In order to evaluate whether FOXG1 could affect the amount of radial glia-like precursor population, we labeled qNSCs with a GFAP tag and FOXG1 with a GFP tag. The GFP^−^GFAP^+^ tag was used to trace the endogenous qNSCs in adult hippocampus. As shown in Figure 2A,E, there is no difference in the endogenous qNSC population between *Foxg1*^fl/fl^ and *Foxg1*^fl/fl^-CreAAV groups. On this basis, we further investigate the amount of total qNSCs (GFAP^+^), a remarkable increase in GFAP^+^ population was observed in FOXG1 overexpressing brains with immunofluorescence (Figure 2A,D,H,J), immunohistochemistry (Figure 2B,F), and with Western blot (Figure 2C,G) analyses. Given that the increase of total qNSCs was accompanied with the similar expression of endogenous qNSCs, the present findings suggest that Cre-induced activation of FOXG1 contributes to newly generate NSCs from the adult hippocampus DG of *Foxg1* knock-in mice. 

To confirm the present results, we traced the proliferated aNSC in adult hippocampal DG. Sections from *Foxg1*^fl/fl^ and *Foxg1*^fl/fl^-CreAAV hippocampus were double immunolabeled for possible coexpression of GFAP and PCNA. Here, we showed that FOXG1 activity contributes to enlarging the aNSC pool, as revealed by an apparent increase in the amount of proliferated aNSCs (PCNA^+^GFAP^+^) in contrast to the control ones (Figure 2H,K). 

qNSC activation is associated with the expression of brain lipid binding protein (BLBP) [17]. Therefore, the activated DG NSCs can be recognized by GFAP^+^BLBP^+^ cells. Quantication analyses revealed a relative elevation in activated DG aNSCs within the GFAP^+^ population in the FOXG1 overexpression brains in contrast to the control ones (Figure 2I,L,M).

### 3.3. FOXG1 Promotes the Formation of Neurosphere and Suppresses p21^cip1^-Mediated Cell Cycle Exit 

**Figure 3:**
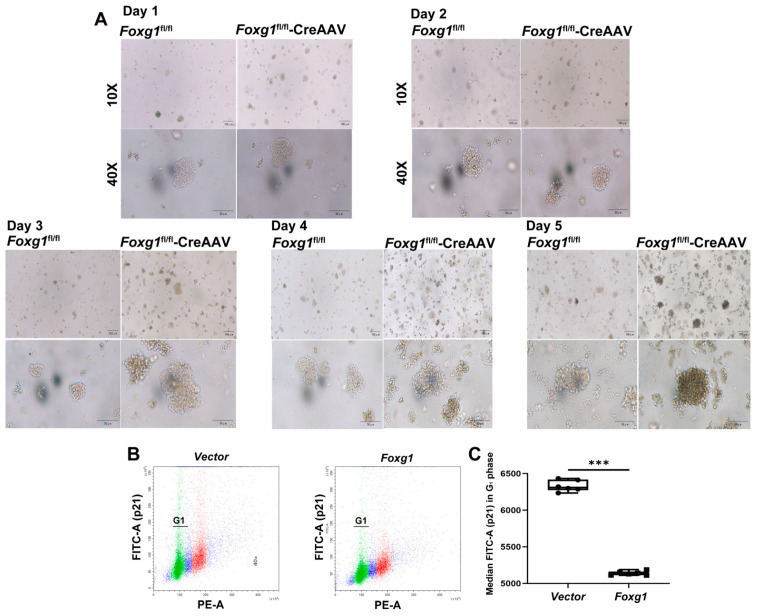
FOXG1 promotes the formation of neurosphere from cultured aNSCs and suppresses p21^cip1^-mediated cell-cycle exit. (**A**) Primary cultures of aNSCs at 1, 2, 3, 4 and 5 days of culture. Scale bar = 100 μm, applies to all images. (**B**) Flow cytometric analysis of p21 positive population in G_1_ phase of cell cycle. Tested N_2_A cells were transfected with *vector* or *Foxg1* plasmids for 48 h. After incubation with an antibody against p21, *vector-* or *Foxg1-*transfected cells were treated with RNase A and propidium iodide mixture. Dot plots show the cell cycle distribution and FITC-A fluorescence intensity. (**C**) Column diagrams show the median fluorescent intensity of FITC-A (the strength of p21 expression) in the G_1_-phase of cell cycle. Values are expressed as means ± S. E. M. For each group, *n =* 6. Significant levels set at *** *p <* 0.001 noted difference between *vector* and *Foxg1* transfected cells.

To investigate the effect of FOXG1 activity on the formation of neurosphere, we first observed the genotype effect on the morphological varies of cultured aNSCs isolated from *Foxg1*^fl/fl^ and *Foxg1*^fl/fl^-CreAAV hippocampus. As compared with the control cells, the cultured neurosphere from the *Foxg1* knock-in mice were well distributed and a few exhibited a proliferative growth style with a transparent cytoplasm during the day 1 (Figure 3A); At day 2–3 of culture, the number of neurosphere increased and the size of cell spheres was uneven. The cytoplasm of all the neurospheres was transparent (Figure 3A). At day 4–5 of culture, the volume of the neurospheres was enlarged in addition to the increased number of cell spheres. At this time, the majority of cells exhibited a suspended growth style, with regular morphology and strong refraction (Figure 3A).

Previous findings have reported that FOXG1-regulated activities enable the expansion of the population of precursor cells likely through suppression of p21^cip1^-mediated cell-cycle exit [5]. Therefore, we investigated p21 positive population by flow cytometry detection. As shown in Figure 3B,C, FOXG1 overexpression obviously reduced the median fluorescent intensity of FITC-A (the strength of p21 expression) in the G_1_ phase, suggesting that the mechanisms of FOXG1 promoting the proliferation of aNSCs is mediated, at least partly, by suppressing p21^cip1^-mediated cell cycle exit. 

### 3.4. FOXG1 Induces aNSCs Giving Rise to Intermediate Progenitor Cells (IPCs) and Enlarges IPC Pool during Precursor Cell Stage

Type 2 cells are characterized by their expression of Eomes (Tbr2), a transcription factor that, during embryonic cortical development, identifies the intermediate progenitor cells (IPCs), which maintain self-renewing properties and can differentiate into neurons [16]

**Figure 4:**
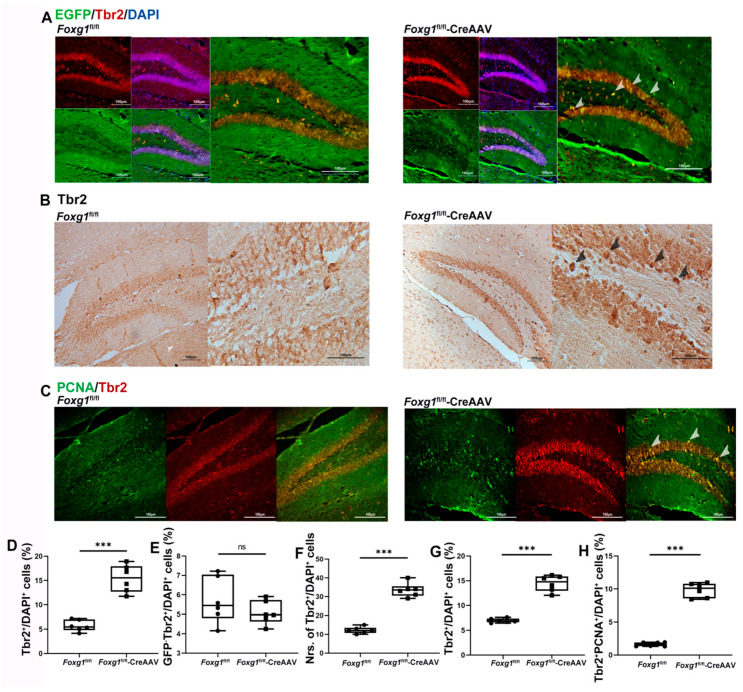
FOXG1 increases the amount of Tbr2-expressing IPCs and promotes their proliferation in adult hippocampal DG of the *Foxg1* genotype mice. (**A**) Expression patterns of Tbr2-expressing IPCs in the hippocampal DG of *Foxg1*^fl/fl^ mice were assessed with immunofluorescence. Red = Tbr2; Green = EGFP-labeled FOXG1; Blue = DAPI. Scale bars = 100 μm. Arrow heads: newly generated IPCs with FOXG1 activation. (**D**) Levels of total intermediate progenitor cells (IPCs) in adult hippocampus are showed as %Tbr2^+^/DAPI^+^ cells. (**E**) Levels of endogenous IPCs in adult hippocampus are showed as %GFP^−^Tbr2^+^/DAPI^+^ cells. (**B**) Expression patterns and (**F**) qualification of total IPCs in adult hippocampus were studied with immunohischemisitry. Scale bars = 100 μm. (**C**) Expression patterns of proliferated IPCs in the hippocampal DG of *Foxg1* genotype mice were assessed with immunofluorescence. Red = Tbr2; Green = PCNA. Scale bars = 100 μm. Arrow heads: proliferated IPCs. (**G**) Levels of IPCs in adult hippocampus are showed as %Tbr2^+^/DAPI^+^ cells. (**H**) Levels of proliferated IPCs in adult hippocampus are showed as %Tbr2^+^PCNA^+^/DAPI^+^ cells. Values are expressed as means ± S. E. M. *n =* 6/group. Significant levels set at *** *p* < 0.001 noted difference between *Foxg1*^fl/fl^ and *Foxg1*^fl/fl^-CreAAV animals. ns—not significant.

To assess the role of FOXG1 in the production of IPCs in adult hippocampal DG, we examined IPC marker Tbr2 in *Foxg1*^fl/fl^ and *Foxg1*^fl/fl^-CreAAV mice. Considering the similar expression of endogenous IPCs (Figure 4A,E), the increased amount of total IPCs (Figure 4A,B,D,F) was caused at least in part by Cre-induced activation of FOXG1, which suggests that FOXG1 contributes to aNSCs giving rise to IPCs in the hippocampus DG.

To confirm that FOXG1 activation promotes proliferation of Tbr2-expressing IPCs in adult hippocampal DG, sections from *Foxg1*^fl/fl^ and *Foxg1*^fl/fl^-CreAAV hippocampus were double immunolabeled for possible coexpression of Tbr2 and PCNA. Here, we showed that FOXG1 activity apparently increased the amount of proliferated IPCs (PCNA^+^Tbr2^+^) in contrast to the control ones (Figure 4C,G,H).

### 3.5. FOXG1 Induces Amplifying Progenitors Giving Rise to Newborn Neuroblasts during Precursor Cell Stage

**Figure 5:**
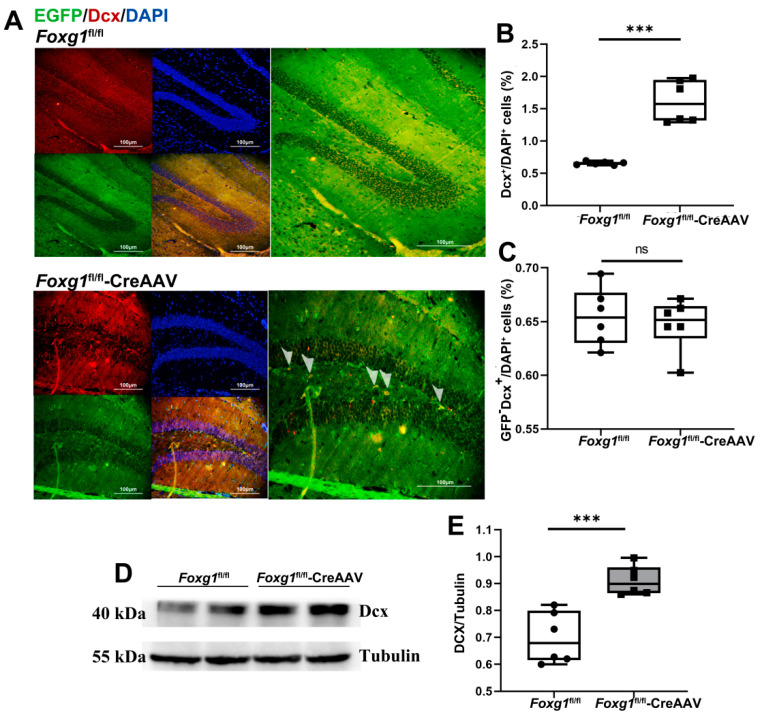
FOXG1 increases the amount of doublecortin (Dcx)-expressing newborn neuroblasts in adult hippocampal DG of the *Foxg1* genotype mice. (**A**) Expression patterns of Dcx-expressing neuroblasts in the hippocampal DG of *Foxg1* genotype mice were assessed with immunofluorescence. Red = Dcx; Green = EGFP-labeled FOXG1; Blue = DAPI. Scale bars = 100 μm. Arrow heads: newly generated neuroblasts with FOXG1 activation. (**B**) Levels of total neuroblasts in adult hippocampus are showed as %Dcx^+^/DAPI^+^ cells. (**C**) Levels of endogenous neuroblasts in adult hippocampus are showed as %GFP^−^Dcx^+^/DAPI^+^ cells. (**D**,**E**) Hippocampal lysates of the two genotype mice were immunoblotted using an antibody against Dcx. Values are expressed as means ± S. E. M. *n =* 6/group. Significant levels set at *** *p* < 0.001 noted difference between *Foxg1*^fl/fl^ and *Foxg1*^fl/fl^-CreAAV animals. ns—not significant.

Among the neuronal lineage markers first appearing at the type 2b stage is Dcx. During hippocampal neurogenesis, the amplifying progenitors give rise to Dcx-expressing neuroblasts. To confirm that the regulating role of FOXG1 in the production of newborn neuroblasts, we assessed total and endogenous amount of Dcx positive cells. Quantification revealed a relative elevation in the numbers of newborn neuroblasts (Dcx^+^) induced by FOXG1 activation within the GFP^+^ population in the *Foxg1* knock-in mice compared to control ones (Figure 5A–C). Consistently, similar results were also confirmed with Western blot analyses (Figure 5D,E).

### 3.6. FOXG1 Affects Cell-Cycle Redistribution 

**Figure 6:**
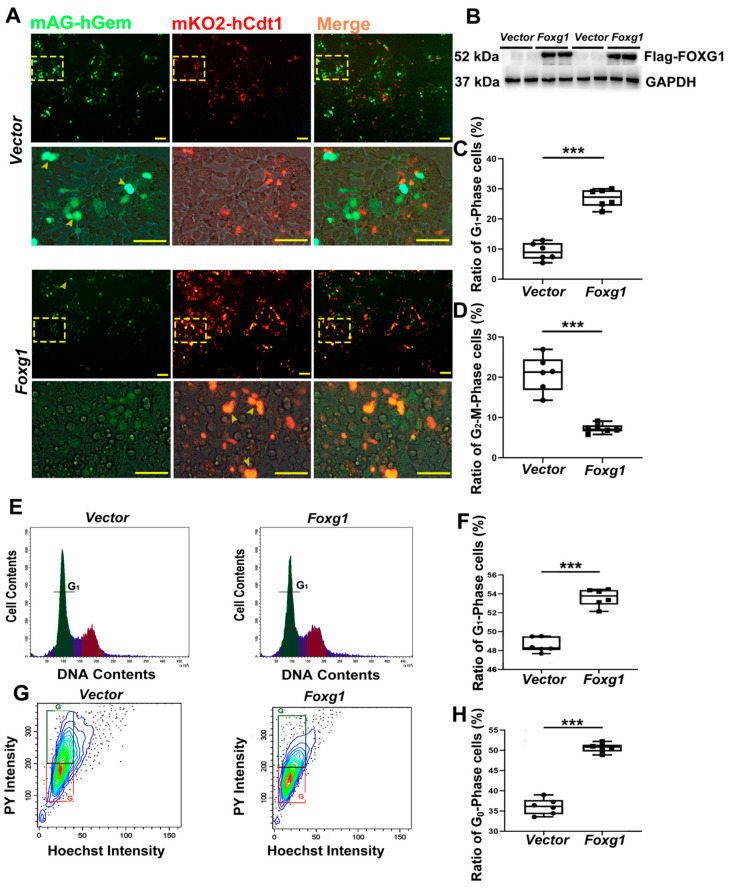
FOXG1 prolongs G_1_-phase length and promotes cell redistribution. FUCCI cells were cultured in 35 mm dishes for 24 h. The cells were transfected with *vector* or *Foxg1* plasmid for 48 h. (**A**,**B**) Fluorescence images of mAG and mKO2 emission signals in *vector* or *Foxg1* transfected 293T cells (Green = mAG; Red = mKO2) were disaplyed. Scale bar = 50 μm. Ratio percent of (**C**) G_1_- and (**D**) G_2_-M phase cells were calculated using the formula [G_1_ phase: mKO2^+^ cells/(mKO2^+^ cells + mAG^+^ cells) × 100%; G_2_-M phase: mAG^+^ cells/(mKO2^+^ cells + mAG^+^ cells) × 100%. (**E**) Flow cytometric analysis of cell cycle distribution. Tested N_2_A cells were treated with RNase A and propidium iodide mixture. Data were gated to distinguish cell cycle. (**F**) Quantitative evaluation of ratio percentage of cells in G_1_-phase. (**G**) N_2_A cells were stained with Hoechst 33342 and pyronin Y (PY). Flow cytometry technology was utilized to analyze cDNA and RNA contents after cells were transfected with *vector* or *Foxg1* plasmids. (**H**) Quantification evaluation of ratio percentage of cells in G_0_-phase. Values are expressed as means ± S. E. M. *n =* 6/group. Significant levels set at *** *p* < 0.001 noted difference between *vector* and *Foxg1* transfected cells.

Recent studies have reported that the increase of G_1_ length of cell cycle would favor stem cell differentiation [18]. To assess the role of FOXG1 in changing the length of the G_1_ phase during precursor cell differentiation, the FUCCI reporter system, which has been shown to be an accurate gauge of cell cycle position [18], was employed in the present study. FUCCI was designed to express mKO2 fused with the ubiquitylation domain of hCdt1, a marker of G_1_ phase, and mAG fused with the ubiquitylation domain of hGem, a marker of G_2_-M phases. We measured firstly the mKO2 traced FUCCI-G_1_ expression by image analysis of fluorescence. This analysis revealed FOXG1 activation increased the G_1_ length of cell cycle, as revealed by more cells expressing FUCCI-G_1_ reporter (mKO2) in *Foxg1* transfected cells in contrast to those in the control ones (Figure 6A–C). 

Further, using the flow cytometry technology, we confirmed that *Foxg1* transfection triggered more cells arrested in G_1_ phase of cell cycle in contrast to the control ones (Figure 6E,F). Given the indication that the prolonged length of G_1_-phase can directly influence the exit of neural precursors from cell-cycle [19]. Therefore, we suggest that FOXG1 activation is beneficial to cells exit from G_1_-phase.

Recent evidence demonstrates that cells entering the G_0_-phase after cell-cycle exit contribute to these cells being either terminally differentiated, senescent, or quiescent [20]. In light of this, the precise control of the cell-cycle exit is essential for the production of differentiated neurons [20]. To explore the role of FOXG1 in regulating cell cycle redistribution, we overexpressed *vector* and *Foxg1* plasmid for 48 h and performed two-dimensional FACS analyses of cellular RNA and DNA contents (Figure 6G,H). *Foxg1* transfection resulted in an increase of cells staining low for pyronin Y (50.69% compared with 37.27% of control cells), which identifies them as quiescent cell populations with low RNA content. A possible explanation for this is that FOXG1 participates in the molecular machinery that coordinates cell cycle exit and the differentiation of neuronal precursors [14].

### 3.7. FOXG1 Promotes Rise of the Final Mature Granule Neurons and Contributes to Synaptic Plasticity during Postmitotic Maturation Phase

**Figure 7:**
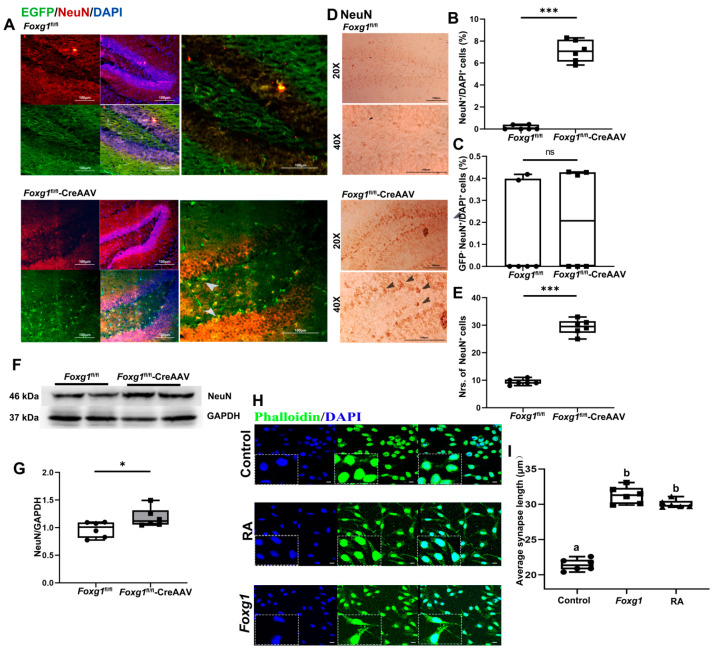
FOXG1 induces final mature of GNs in adult hippocampus DG and increases synaptic plasticity. (**A**) Expression patterns of NeuN-expressing GNs in the hippocampal DG of *Foxg1* genotype mice were assessed with immunofluorescence. Red = NeuN; Green = EGFP-labeled FOXG1; Blue = DAPI. Scale bars = 100 μm. Arrow heads: newly generated GNs with FOXG1 activation. (**B**) Levels of total GNs in adult hippocampus are showed as %NeuN^+^/DAPI^+^ cells. (**C**) Levels of endogenous GNs in adult hippocampus are showed as %GFP^−^NeuN^+^/DAPI^+^ cells. (**D**) Expression patterns and (**E**) qualification of total GNs in adult hippocampus were studied with immunohischemisitry. Scale bars = 100 μm. (**F**,**G**) Hippocampal lysates of the two genotype mice were immunoblotted using an antibody against NeuN. GAPDH was used as the loading control. Values are expressed as means ± S. E. M. For each group, *n =* 6. Significant levels set at * *p* < 0.05, *** *p* < 0.001 noted difference between *Foxg1*^fl/fl^ and *Foxg1*^fl/fl^-CreAAV animals. (**H**) Fluorescence microscopic analysis of differentiation-related morphological changes of N_2_A cells by stained the F-actin cytoskeleton with fluorescently labeled phalloidin. Green = Phalloidin; Blue = DAPI. As positive control, cells were differentiated with 10 μM RA. Scale bars = 25 μm. (**I**) Quantification of the average neurite lengths are illustrated in the column diagrams. Values are expressed as means ± S. E. M. For each group, *n =* 6. Different letters indicate statistical differences in mean values among groups (*p* < 0.05). ns—not significant.

Dcx extends from a proliferation stage, through cell cycle exit, to a period of postmitotic maturation that lasts 2–3 weeks [16]. When neuroblasts exit the cell-cycle, they will differentiate into final mature granule neurons (GNs). Given that GNs express neuronal nuclear marker NeuN, Prox-1, calbindin, and β-III tubulin [21], we evaluated the effect of FOXG1 activation on the production of NeuN-expressing GNs. As shown in Figure 7A–E, FOXG1 activation induced NSCs differentiated into mature neuron, as revealed by an increased NeuN positive population in the adult hippocampus of *Foxg1* knock-in mice. 

Within days after cell-cycle exit, morphological maturation of the newborn GNs is revealed as the most visible expression in the extension of the dendrites [16]. Therefore, by staining the F-actin cytoskeleton with fluorescently labeled phalloidin and manually quantifying the average neurite length, we visualized the effect of FOXG1 activity on differentiation-related morphological changes in cells. As a reference, 3 d treatment with RA induced the expected phenotype of N_2_A cells with the formation of extensive neurite network. As displayed in Figure 7H,I, a remarkable increase in neurite length was observed in *Foxg1* transfected cells when compared to the control ones. Notably, it seemed to attain a complete RA-induced morphology, which suggests that FOXG1 contributes to increasing synaptic plasticity. 

### 3.8. FOXG1 Can’t Induce the Production of Oligodendrocytes

Research over the last decade has revealed that aNSCs are operationally defined as multipotent, self-renewing cells in the CNS that are characterized by their ability to differentiate into neuron, astrocyte, and oligodendrocytes via gene regualtion [22]. 

**Figure 8:**
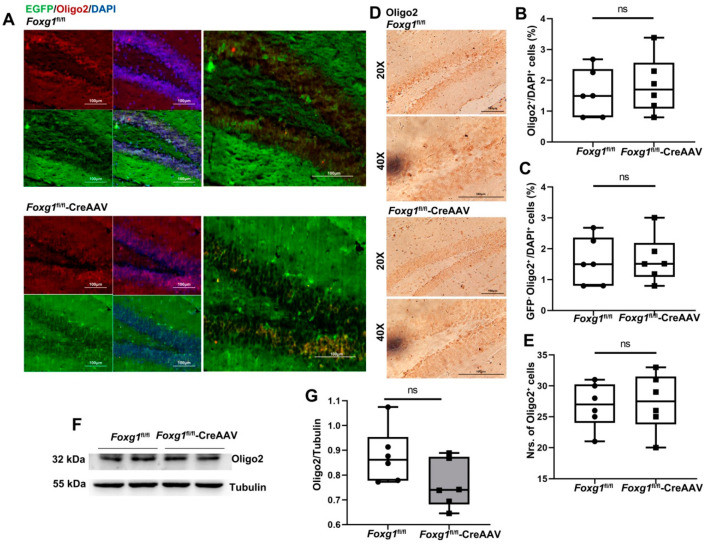
FOXG1 has no ability to induce the production of oligodendrocytes in adult hippocampal DG of the *Foxg1* genotype mice. (**A**) Expression patterns of oligodendrocytes in the hippocampal DG of *Foxg1* genotype mice were assessed with immunofluorescence. Red = Oligo2; Green = EGFP-labeled FOXG1; Blue = DAPI. Scale bars = 100 μm. (**B**) Levels of total oligodendrocytes in adult hippocampus are showed as %Oligo2^+^/DAPI^+^ cells. (**C**) Levels of endogenous oligodendrocytes in adult hippocampus are showed as %GFP^−^Oligo2^+^/DAPI^+^ cells. (**D**) Expression patterns and (**E**) qualification of total oligodendrocytes in adult hippocampus were studied with immunohischemisitry. Scale bars = 100 μm. (**F**,**G**) Hippocampal lysates of the two genotype mice were immunoblotted using an antibody against Oligo2. Tubulin was used as the loading control. Values are expressed as means ± S. E. M. *n =* 6/group. ns—not significant.

To evaluate whether FOXG1 contributes to precursor cells giving rise to oligodendrocytes, we traced oligodendrocytes with Oligo2 tag. As shown in Figure 8A–G, FOXG1 activation has no effect on the Oligo2 positive population, suggesting that FOXG1 could induce aNSCs to differentiate into mature neurons, but not into oligodendrocytes during adult neurogenesis. 

## 4. Discussion

Accumulate evidence indicates that FOXG1 plays important roles in pattern formation, cell proliferation, and cell specification during embryonic development [5]. FOXG1 was recently identified as strongly expressed in the hippocampal DG, a specific region involved in neurogenesis throughout life in adult brain [23]. Thus far, the role of FOXG1 in adult neurogenesis and its underlying mechanisms remain largely unknown.

In a previous attempt to determine the role of FOXG1 in fate acquisition of DG neurons during hippocampal neurogenesis, the Schäffner lab (2022) isolated aNSCs from hippocampus DG of wild type mice, and established a FOXG1-overexpressing in vitro system by utilizing retrovirus-mediated *Foxg1* transduction [24]. The broad deficits concerning the in vitro studies are that they are unable to see how the body as a whole will respond to a particular stimulus in contrast to an in vivo system. To overcome this issue, the current study introduced a specific *Foxg1* “knock-in” (CAG-loxp-stop-loxp-Foxg1-IRES-EGFP (*Foxg1*^fl/fl^)) mouse line, which could be used to precisely activates FOXG1 in hippocampal DG with CreAAV infusion. Using the FOXG1-overexpressing in vivo system, we examine the effect of FOXG1 on neuronal lineage progression and its potential mechanism with a particular focus on adult hippocampal neurogenesis.

Adult hippocampal neurogenesis is a multistep process that originates from a sequence of proliferative precursor cells. On the precursor cell level, the cascade originates in a radial glia-like cell or type I (also termed as aNSC). In contrast to embryonic neurogenesis, aNSCs are mostly quiescent with only a few progressing through the cell cycle [21]. They are generally identified by GFAP, specific molecular markers of qNSCs [25]. In this study, we demonstrated that FOXG1 signaling is required for enlarging aNSC pool, as revealed by an increase in the amount of PCNA^+^GFAP^+^ cells in the hippocampal DG of *Foxg1* “knock-in” mouse, as well as the promotion of neurosphere-forming under in vitro culture condition. Consistently, at prenatal and early postnatal stages, a remarkable reduction in the numbers of NSCs in the DG was reported in *Foxg1* conditional knockout mice [26], suggesting that FOXG1 contributes to the self-renewal of NSCs not only at prenatal and early postnatal stages, but also at adult stage. On that basis, we next asked the question how this function is achieved. Being one of the important cell cycle inhibitors, p21 plays a major role in maintaining the quiescence of adult neural precursors. The depletion of this factor leads to a transient activation of precursor pool [27]. We [28] and others [5] have shown that FOXG1 is involved in the suppression of p21 expression. The present data are consistent with the above reports. Besides, we additionally confirmed that FOXG1-regulated activity enables the expansion of the aNSC population, likely through lowering the p21^cip1^ expression during the G_1_-phase of cell cycle.

Given that GFAP is not a valid marker since it also expressed by parenchymal astrocytes, whose expression may be upregulated by the gliosis typically associated to brain injury or insult [29], and that GFAP could be a valid marker only when combined with additional specific markers of the type I progenitors, such as Sox2 or BLBP [16]. Therefore, we evaluated the numbers of GFAP^+^ BLBP^+^ population and confirmed that FOXG1 contributes to aNSC activation (GFAP^+^ BLBP^+^ cells) in the hippocampal DG of *Foxg1* “knock-in” mouse.

Upon activation, aNSCs predominantly divide asymmetrically to produce another NSC and a type II cell. Type II progenitors represent an important stage of clonal expansion and linage choice [21]. Phenotypically, these intermediate cells are characterized by their expression of Eomes (Tbr2) [16]. Therefore, the effect of FOXG1 on the production of the basal progenitors (GFP^+^ Tbr2^+^ cells) and the proliferated IPCs (PCNA^+^ Tbr2^+^ cells) was examined in the present study. Forced expression of FOXG1 enlarged the IPC pool in the adult hippocampus DG of *Foxg1* knock-in brain. This, combined with another report about reducing IPC numbers observed in the hippocampus of *Foxg1* knock-out mice at the prenatal and the early postnatal stages [26], has led to the suggestion that FOXG1 plays a crucial role in self-renewing and differentiation of IPCs both in prenatal and in adult stage. 

Among the neuronal lineage markers first appearing at the type IIb stage is Dcx [30]. Dcx is widely used as a stage-specific marker that extends from a proliferation stage (amplifying progenitor cells, type IIb), through cell cycle exit (migrating neuroblasts, type Ⅲ), to a period of postmitotic maturation [30]. Therefore, the possibility of FOXG1 in promoting the production of Dcx-labeled neuroblasts was evaluated, and the results we obtained strongly suggested that the FOXG1 activation promotes the differentiation of aNSCs giving rise to immature neurons. 

A number of studies have indicated that the length of G_1_-phase can directly influence the differentiation of neural precursors [19]. However, the role of FOXG1 in regulating cell cycle exit in adult neurogenesis is largely unknown. Here, we introduced a FUCCI reporter system to accurate gauge cell cycle position and assessed the role of FOXG1 in regulating the length of G_1_-phase during precursor cell differentiation. The present results indicate that activation of FOXG1 signaling delays G_1_-S phase transition in vitro. Given that *Foxg1* is responsible in positively regulating Pax6 transcription [31], as well as the function of Pax6 in delaying cdks/cyclins-dependent cell cycle length [19], the function of FOXG1 on accelerating lineage-committed cells exit and differentiation could be mediated, at least partly by the upregulation of Pax6/cdks pathway. 

In adult SGZ, the newborn, lineage-committed neuroblasts exit the cell cycle and enter a maturation stage, during which they extend their dendrites into a molecular layer, their axon to CA_3_, thereby increasing synaptic plasticity [27]. We examined the role of FOXG1 signaling in regulating the production and differentiation of DG cells in the adult hippocampus. FOXG1 activation promotes maturation of adult-born neurons as revealed by a strong increase in NeuN-expressing DG cells. This, along with the finding from an in vitro study [32] that *Foxg1* conditional knockout from DG disrupts maturation of postmitotic neuron during prenatal and postnatal development [26], has led to the suggestion that FOXG1 activation contributes to not only prenatal, but also adult hippocampal neurogenesis. Besides, we observed the morphological changes of neurite outgrowth in N_2_A cells. Compared with few and shorter neurite-like extensions displayed in undifferentiated control cells, *Foxg1* transfection prominently enhanced neurite outgrowth, which is in line with the description that Lentiviral-mediated *Foxg1* delivery dramatically stimulates neurite outgrowth in cultured NSCs [32]. 

Recent research indicates that *Foxg1* knockout decreases the proliferation of oligodendrocyte precursor cells and accelerates their differentiation into mature oligodendrocytes both in vivo and in vitro [33]. As such, we questioned whether *Foxg1* knock-in would affect the expression of Oligo2-expressing oligodendrocytes. Notably, no obvious varies of the numbers of oligodendrocytes were found in the adult hippocampal DG of *Foxg1* knock-in brain. The discrepancy effect of FOXG1 on the production of Oligo2^+^ population is caused, at least in part, by the mutation position of those genotype mice. In an attempt to determine the role of FOXG1 in demyelination and remyelination in the brain, the Fuxing lab generated a *Foxg1* knock-out mouse line through the transgenic expression of Cre recombinase under the control of nestin *(Nes)* promoter [33]. These reported mice effectively deplete *Foxg1* gene in both the central and peripheral nervous system, including neuronal and glial cell precursor [33]. In the present study, however, FOXG1 was chosen for overexpression in the adult hippocampal DG with CreAAV infusion to elucidate the possible relevance of FOXG with adult neurogenesis. 

## 5. Conclusions

Taken together, the present result indicates that FOXG1 induces aNSCs, giving rise to IPCs, neuroblasts in the hippocampal DG. FOXG1-regulated activities enable the expansion of the precursor cell pool by inhibiting p21^cip1^ expression. Through increasing the length of G_1_-phase, FOXG1 promotes lineage-committed cells to exit the cell cycle and differentiate into final mature GNs. Therefore, by inducing the activation of FOXG1, a de novo production of neurons in the DG region has introduced the possibility of a new form of plasticity that could sustain memory processes. 

## Data Availability

The data that support the findings of this study are available from the corresponding author upon reasonable request.
